# Gene-based SNP discovery and genetic mapping in pea

**DOI:** 10.1007/s00122-014-2375-y

**Published:** 2014-08-15

**Authors:** Anoop Sindhu, Larissa Ramsay, Lacey-Anne Sanderson, Robert Stonehouse, Rong Li, Janet Condie, Arun S. K. Shunmugam, Yong Liu, Ambuj B. Jha, Marwan Diapari, Judith Burstin, Gregoire Aubert, Bunyamin Tar’an, Kirstin E. Bett, Thomas D. Warkentin, Andrew G. Sharpe

**Affiliations:** 1 Department of Plant Sciences, Crop Development Centre, University of Saskatchewan, 51 Campus Drive, Saskatoon, SK S7N 5A8 Canada; 2 National Research Council Canada, 110 Gymnasium Place, Saskatoon, SK S7N 0W9 Canada; 3 UMR1347 Agroecology, INRA, 17 rue de Sully, 21065 Dijon Cedex, France; 4 Present Address: Department of Plant Sciences, Crop Development Centre, University of Saskatchewan, 51 Campus Drive, Saskatoon, SK S7N 5A8 Canada

## Abstract

****Key message**:**

**Gene-based SNPs were identified and mapped in pea using five recombinant inbred line populations segregating for traits of agronomic importance.**

**Abstract:**

Pea (*Pisum sativum* L.) is one of the world’s oldest domesticated crops and has been a model system in plant biology and genetics since the work of Gregor Mendel. Pea is the second most widely grown pulse crop in the world following common bean. The importance of pea as a food crop is growing due to its combination of moderate protein concentration, slowly digestible starch, high dietary fiber concentration, and its richness in micronutrients; however, pea has lagged behind other major crops in harnessing recent advances in molecular biology, genomics and bioinformatics, partly due to its large genome size with a large proportion of repetitive sequence, and to the relatively limited investment in research in this crop globally. The objective of this research was the development of a genome-wide transcriptome-based pea single-nucleotide polymorphism (SNP) marker platform using next-generation sequencing technology. A total of 1,536 polymorphic SNP loci selected from over 20,000 non-redundant SNPs identified using deep transcriptome sequencing of eight diverse *Pisum* accessions were used for genotyping in five RIL populations using an Illumina GoldenGate assay. The first high-density pea SNP map defining all seven linkage groups was generated by integrating with previously published anchor markers. Syntenic relationships of this map with the model legume *Medicago truncatula* and lentil (*Lens culinaris* Medik.) maps were established. The genic SNP map establishes a foundation for future molecular breeding efforts by enabling both the identification and tracking of introgression of genomic regions harbouring QTLs related to agronomic and seed quality traits.

**Electronic supplementary material:**

The online version of this article (doi:10.1007/s00122-014-2375-y) contains supplementary material, which is available to authorized users.

## Introduction

Pea (*Pisum sativum* L., 2*n* = 2*x* = 14) belongs to the Fabaceae family of flowering plants which includes other important agricultural crops such as soybean, chickpea, lentil, alfalfa, peanut, and common bean. Being a rich source of protein, polysaccharides and providing slowly digestible starch, soluble sugars, fiber, minerals, and vitamins, pea plays an important role in meeting human nutritional needs (Smýkal et al. 2012). In addition to the use of mature seeds for food and feed, the pea crop can be used as a vegetable, forage, silage, or green manure. Like other legumes, pea contributes to the development of low-input farming systems by fixing atmospheric nitrogen, and serves as a break crop which reduces the need for pesticide inputs. Global dry pea cultivation over the past decade has ranged from 6.0 to 6.5 million hectares producing 9.4–11.7 million tons per year (FAOSTAT 2013). In addition, global cultivation of vegetable pea has ranged from 1.6 to 2.2 million hectares producing 12.0–17.4 million tons per year over the past decade (FAOSTAT 2013).

Genetic studies in pea have a long history, starting from the early 1790s (Thomas Andrew Knight, as indicated in Ellis 2009) to the very birth of the principles of genetics (Mendel 1865). Yet, despite being an important crop for global food security, pea has not received as much attention as other crops and has lagged behind in the development of molecular biology, genomics and bioinformatics tools (Bhattacharyya et al. 1990; Hofer et al. 2009). Along with the high cost of developing genomic resources, its large genome size (~4 Gbp; Arumuganathan and Earle 1991), recalcitrance to transformation (Clemow et al. 2011), and lack of a reference genome sequence have been the major contributing factors hindering the development of genomic resources in pea. Thus, despite the early lead, pea has lagged behind other major crops (including wheat, rice, corn, and soybean) in terms of genomic resource development.

Developing a genome-wide arrayed class of molecular markers and generating saturated molecular maps are among the first steps in the application of molecular breeding, genomic selection, tagging and gene introgression strategies (Timmerman-Vaughan et al. 2004; Burstin et al. 2007; Lejeune-Hénaut et al. 2008). Despite being a crop species with a large genome, pea is one of the few crop species where early genetic maps were developed using phenotypic and physiological characteristics (Blixt 1974), isozymes (Weeden et al. 1998), and early DNA-based markers (Weeden et al. 1998; Ellis et al. 1992). A pea linkage map consisting of 209 markers spanning 1330 cM was developed using AFLP, RFLP, and RAPD markers (Gilpin et al. 1997), and a consensus map derived from several segregating populations defining seven linkage groups using a combination of morphological, biochemical, and molecular markers was developed by Weeden et al. (1998).

Most of the existing markers are non-targeted (anonymous) genomic markers useful in providing a framework for genetic map development and in assessing genetic diversity, germplasm characterization and have also been used in mapping of quantitative trait loci (QTLs). However, since they are usually not part of the expressed portion of the genome, they are likely to provide, at most, indirect information on functional genes (van Treuren and van Hintum 2009). Therefore, attempts have been made to generate genic molecular markers in pea such as EST-PCR based (Ellis and Poyser 2002; Miesel et al. 2005; Aubert et al. 2006; Bordat et al. 2011), EST-derived SSR (Burstin et al. 2001; Smýkal et al. 2008; Gong et al. 2010; De Caire et al. 2011), and intron targeted markers (Brauner et al. 2002) for the construction of genetic maps. Since transcriptomic markers are derived from gene sequences, they are also useful for establishing syntenic relationships with related crop species.

With the advent of high-throughput genotyping technologies, single-nucleotide polymorphisms (SNPs) have become the marker of choice due to their abundance and the availability of high-throughput screening techniques. In humans, their frequency is about one SNP in every 1000 bp while in soybean on average one SNP occurs in every 200–300 bp, with intronic regions showing three times higher frequency than exonic regions (Wang et al. 1998; Zhu et al. 2003). Development of several platforms for detecting SNPs has led to the refinement of the technology and now genome-wide SNP databases can be generated at reduced cost and in a much shorter time compared to 10 years ago. Many crop species now have high-density SNP maps available which have proven to be valuable resources for practical applications in gene mapping, association mapping, and genomic selection (Jing et al. 2007; Vignal et al. 2002; Varshney et al. 2005). Until recently in pea, however, only limited SNP resources have been available (Aubert et al. 2006; Deulvot et al. 2010). SNP discovery and mapping carried out by Bordat et al. (2011), using 214 EST-derived markers, resulted in a resource to deploy in silico mapping of 5,460 pea unigenes using genomic resources developed in the model legume *Medicago truncatula*. More recently, it has been possible to generate high-throughput de novo transcriptome sequence data in pea using next-generation sequencing (NGS) technology (Franssen et al. 2011; Kaur et al. 2012). The transcriptome sequencing approach provides transcript sequences that are much shorter in length than the ESTs generated using Sanger sequencing, however, these deep data sets serve as a good resource for gene expression analysis, metabolic pathway and cellular process analysis as well as the detection and mining of genic SNPs, as has been described recently in pea (Leonforte et al. 2013; Duarte et al. 2014), as well as in other pulse crops such as lentil (Sharpe et al. 2013). Leonforte et al. (2013) described a set of 408 gene-based SNPs identified between two pea genotypes which were placed in an SSR-anchored genetic map that enabled comparative analysis with model legume genomes as well as the identification of QTLs for salt tolerance.

The objective of this research was the development of a transcriptome-based SNP marker resource in pea. We used targeted 3′end cDNA sequencing of eight *Pisum* accessions for SNP discovery and developed a dense consensus genetic map by genotyping five diverse recombinant inbred line (RIL) populations.

## Methods

### Plant material

Eight diverse pea accessions were utilized in this research including six *Pisum sativum* cultivars (CDC Bronco, Alfetta, Cooper, CDC Striker, Nitouche, and Orb) and two wild accessions P651 (*P. fulvum*), PI 358610 (*P. sativum* ssp. *abyssinicum*) (Table 1). The cultivars were developed in Canada or Western Europe and all are adapted to North American production, while the *P. sativum* subspp. *abyssinicum* and *P. fulvum* accessions were sourced from germplasm collections in USA and Spain, respectively (Table 1). The *P.* *sativum* subspp. *abyssinicum* accession (PI 358610) is a land race collected in Ethiopia (http://www.ars-grin.gov/cgi-bin/npgs/html/taxon.pl?28663). CDC Bronco (Warkentin et al. 2005) was used as the reference genome relative to which SNPs were called as it is a widely grown cultivar in Canada and is one of the parents of several RIL populations developed at the University of Saskatchewan. Line 1-2347-144, a parent in RIL population PR-15, is derived from mutagenesis of CDC Bronco, and retains the majority of the CDC Bronco traits (Warkentin et al. 2012) (Table 2). Nitouche and PI 358610 (*P. sativum* ssp. *abyssinicum*) were not parents of any of the RIL populations but were included in this research to increase genetic diversity. A single plant of each genotype was selfed to produce enough seed for tissue collection and library preparation. Leaf, stem, flowers and developing seeds were collected from plant materials grown in a growth chamber with 16 h light/8 h dark with day temperature 23 °C and night temperature 15 °C. Additional samples were collected from etiolated seedlings grown on filter paper soaked with sterile water in sealed Petri plates with no light. Five RIL populations, derived from diverse parents, were used for genetic mapping. All except Pop9 are from the University of Saskatchewan field pea breeding program and were developed for the purpose of mapping QTLs for traits of importance including disease resistance and nutritional value. These populations were developed by selfing using the single seed descent method from the F_2_ generation, then seeds from single F_7_ plants were bulked. Summarized RIL population parentage and their origins are provided in Supplementary Table S1. In total 586 RILs from five populations were used for mapping. DNA was extracted using a modified CTAB method (Doyle and Doyle 1990) from freeze-dried leaf tissue collected from at least five plants of each RIL to try to assess residual heterozygosity within each RIL.

**Table 1:** SNP discovery results in seven pea (*Pisum*) accessions against the reference cultivar CDC Bronco

Accession	Species	Origin	Total 454 reads	Reference assembly		Contigs with SNPs	Total SNPs^a^	Average read depth
CDC Bronco	*P. sativum*	CDC, Canada	520,797	29,725		N/A	N/A	N/A
Alfetta	*P. sativum*	Limagrain, Netherlands	589,724	N/A		2,797	7,532	6
Nitouche	*P. sativum*	DLF Trifolium, Denmark	593,297	N/A		2,984	7,993	6
Cooper	*P. sativum*	Limagrain, Netherlands	584,720	N/A		3,252	8,723	6
CDC Striker	*P. sativum*	CDC, Canada	537,572	N/A		2,777	7,247	5
Orb	*P. sativum*	Sharpes International, UK	593,701	N/A		2,712	6,881	5
PI 358610	*P. sativum*, subspp. *abyssinicum*	USDA, *Pisum* collection	540,828	N/A		5,807	20,424	5
P651	*P. fulvum*	IFAPA, Spain	574,009	N/A		6,180	24,591	5
Total			4,008,648			26,509	83,391	
Total NR^b^						6,701	20,008	

*CDC* Crop Development Centre, University of Saskatchewan, Saskatoon, Canada; *USDA* United States Department of Agriculture, Pullman, WA, USA; *IFAPA* Instituto de Investigacion y Formacion Agraria y Pesquera, Spain

^a^Number of high-quality SNPs detected in each genotype

^b^Total non-redundant set of contigs with homology to either Medicago or *Glycine max* genome and carrying high-quality SNPs (see also Supplementary Table 2)

**Table 2:** RIL populations, SNP polymorphism, mapped markers and markers shared by each population in consensus map

Population	RIL size	Poly-morphic SNPs	Mono-morphic	Dominant	Failed/ unscorable	Mapped SNPs (with SSR^a^)	No. of markers shared per pair of RIL population in consensus map			
							PR-02	PR-07	PR-15	PR-19
PR-02 Orb × CDC Striker^b^	90	340	1,029	7	160	308				
Pop-9 Cameor × China^c^	124	405 (499^a^)	992	42	97	391 (485^a^)	138	103	125	109
PR-19 Alfetta × P651 (*P. fulvum*)^d^	144	940	457	49	90	303	105	57	88	
PR-15 1-2347-144 × CDC Meadow^e^	94	341	1,019	4	172	308	124	100		
PR-07 Carerra × CDC Striker^b^	134	388	1,043	22	83	245	119			

^a^Including previously published 94 frame-work markers

^b^Warkentin et al. (2004); see also Suppl. Info

^c^Bordat et al. (2011); see also Suppl. Info

^d^Fondevilla et al. (2005); Jha et al. (2012); see also Suppl. Info

^e^Warkentin et al. (2012); see also Suppl. Info

### 3′ anchored cDNA library construction and sequencing

Five kinds of tissue samples were collected from each accession including leaf (2 week old), stem (before flowering), etiolated seedlings (1 week old), flower (mixed stages) and developing seeds (mixed stages). RNA extraction, 3′-cDNA library construction, and 454 Roche Titanium sequencing were carried out exactly as described in Sharpe et al. (2013). Briefly, total RNA from leaves was extracted using the RNeasy Plant Mini Kit (Qiagen) including on-column DNase digestion. Total RNA from other tissues was extracted using the CTAB method described by Miesel et al. (2005) and then cleaned up using RNeasy Mini kits (Qiagen), including on-column DNase digestion. 3′-anchored cDNA libraries for 454 sequencing were prepared based on a protocol described in Eveland et al. (2008) and modified to incorporate *Aci*I as the restriction enzyme used to generate 3′ cDNA fragments of the optimal size range for amplification during 454 Titanium chemistry sequencing (Parkin et al. 2010). The *Aci*I-digested cDNA was treated with Agencourt AMPure Beads (Beckman Coulter Inc.) to remove smaller fragments (<250 bp). The 3′-fragments of cDNA were recovered using DynBeads M-270 streptavidin (Invitrogen) and then ligated with A-adaptor. Roche 454 Titanium sequencing of the titrated single strand DNA libraries was carried out following the procedure described by Margulies et al. (2005) with modifications for the Titanium chemistry as described in protocols supplied by the manufacturer (Roche, Laval, Quebec).

### Sequence assembly, analysis and SSR analysis

A de novo assembly of the CDC Bronco 454 reads was performed using NGen (DNAStar) software as described in Sharpe et al. (2013) with a few differences as follows. Parameters used for the de novo assembly included: Min/Match Percent = 90; Max 454 Sequence Length = 600; Repeat Handling On; and Expected Coverage = 20. Processing was carried out on a Dell R910, 2 × 2.40 GHz, 48 GB RAM Windows 64 Bit server. Following assembly, repeat class contigs were removed as well as any remaining contigs of <200 bp. Sequence data from the other accessions were then assembled against the CDC Bronco de novo reference assembly using NGen (DNAStar). To ensure removal of the 454 key sequence from reads, 10 bp were trimmed from the 5′ ends. Adapter screening was used to remove the wobble primer, the adapter, and any poly-A tail. Contaminant filtering was implemented using a set of four mitochondrial, chloroplast, and ribosomal sequences.

The identification of candidate SSRs in the reference assembly together with numbers of potentially polymorphic SSR loci amongst the *P. sativum* and *P. fulvum* accessions was carried out using the software QDD (Meglécz et al. 2010). The primer pairs flanking these loci were also generated using QDD.

### SNP reporting

Single-nucleotide polymorphisms present in the seven accessions relative to CDC Bronco were identified using Seqman Pro (DNAStar) as described in Sharpe et al. (2013) using the CDC Bronco assembly as a reference. Individual reports for each accession were parsed into spreadsheet format for comparison using a custom Perl pipeline. Only transition and transversion SNPs were reported; indels were ignored as the nature of the pyrosequencing reduces the robustness of called indels (Barbazuk et al. 2007). The final spreadsheet report (Supplementary Table S2) indicates if the SNP is the same as the reference, the alternate allele, or if there is no sequence data at that position. All low-confidence SNPs (represented as <80 % of aligned reads have the called SNP or <3 aligned reads in total with called SNP) were identified and reported as being below thresholds if found in the same position as confident SNPs.

The 454 contigs were initially mapped against the annotated *Medicago* and soybean genomes with BLAT (Kent 2002), followed by a secondary mapping as described in Sharpe et al. (2013) using GMAP (Wu and Watanabe 2005) with the cross-species parameter for potential gene duplicate identification. Flanking sequence length and gene annotation information (Supplementary Table S2) were extracted from the sequence mapping output via a custom Perl script.

### SNP validation

A subset of 32 SNPs, identified from the 454 sequence alignments, was selected for validation in silico by comparison to individual raw 454 reads, and through the development of KASP SNP assays (KBioscience, Hoddeston, UK). The SNPs were selected to assess a variety of different levels of sequence read depth, variable allele frequency, the presence or absence of SNPs in sequence flanking targeted SNPs, and marker quality score (Illumina assay design tool (ADT) score). ADT score predicts success information, validation status, and minor allele frequencies. Single SNP validation was carried out using in-house developed KASP assays as described by Sharpe et al. (2013) on the six *P. sativum* cultivars used for SNP discovery.

### Illumina GoldenGate OPA design

From the 8,822 target SNPs identified in 4,194 contigs and selected for potential array development, an additional filtering step was employed to remove potentially heterozygous SNPs by identifying those SNPs that showed less than 100 % of the reads matching either the reference or the alternate allele (1,018 SNPs; Supplementary Table S2). These SNPs were removed to avoid potential issues with assay reliability for genotyping in targeted RILs. Sequence data for the contigs surrounding the SNPs were checked for the number of base pairs to the end of the contig, to the next SNP, or to the closest splice site. Those with less than 60 bp in this flanking region were eliminated since they cannot be used for Illumina GoldenGate arrays (Illumina Inc., San Diego, CA, USA). All remaining candidate SNPs were submitted to Illumina for assay design and a total of 7,229 SNPs were returned with ADT rank scores that indicate the likelihood of assays working based on an ideal score of 1 and acceptable to utilize with a minimum score of 0.4; preferential selection was given to ones scoring above 0.6 as recommended by Illumina. A filtered set of 3,106 SNPs, in which only the designed SNP marker with the highest ADT score per contig, was retained. Since some contigs overlapped at the same orthologous gene mapping position in *Medicago*, a filtered set of 2,646 SNPs, where such position duplicates had been removed, was selected. From these, all SNPs associated with a GoldenGate assay ADT score below 0.4 were removed leaving a total of 2,594 SNPs for final selection based upon observed allelic variation amongst the six *P. sativum* cultivars and a minimal amount of missing data. In total 1,245 SNPs showed a polymorphism among the cultivars, but 138 of these had large amounts of missing data leaving 1,107 selected for the GoldenGate Oligo Pool Assay (OPA) synthesis. From the remaining 1,349 SNPs that did not show any differences among the cultivars, but were polymorphic between either one or both of the *P. sativum* ssp. *abyssinicum* and *P. fulvum* accessions and the reference CDC Bronco, a set of additional markers was chosen. Starting with missing data from just one genotype, additional markers were chosen that had the highest ADT score and a reasonably low amount of missing data (up to four genotypes with missing data). This resulted in 429 additional markers providing a final set of 1,536 SNPs represented on the pea GoldenGate array (Ps1536 OPA; Supplementary Table S2).

### SNP genotyping and construction of consensus pea genetic map

A total of 586 RILs from five mapping populations (Table 2; Supplementary Table S3) were genotyped using the Ps1536 OPA using a standard GoldenGate protocol following the instructions provided by the supplier (http://www.illumina.com/technology/goldengate_genotyping_assay.ilmn). The PCR products generated were scanned for genotying using an Illumina HiScan (Illumina Inc., San Diego, CA, USA). GenomeStudio software 2010.3 (Illumina Inc., San Diego, CA, USA) was used for data clustering and allele calls were visually inspected for errors in automatic allele calling and corrected where deemed necessary. Any calls that were not clearly one allele or the other were reported as missing data to avoid errors. The segregation data in individual RIL populations were subjected to Chi square test to determine deviations from balanced segregation ratios. The markers showing Chi square values more than five were not used, except in the PR-19 RIL population where a higher degree of segregation distortion was present and Chi square values were higher.

**Fig. 1:**
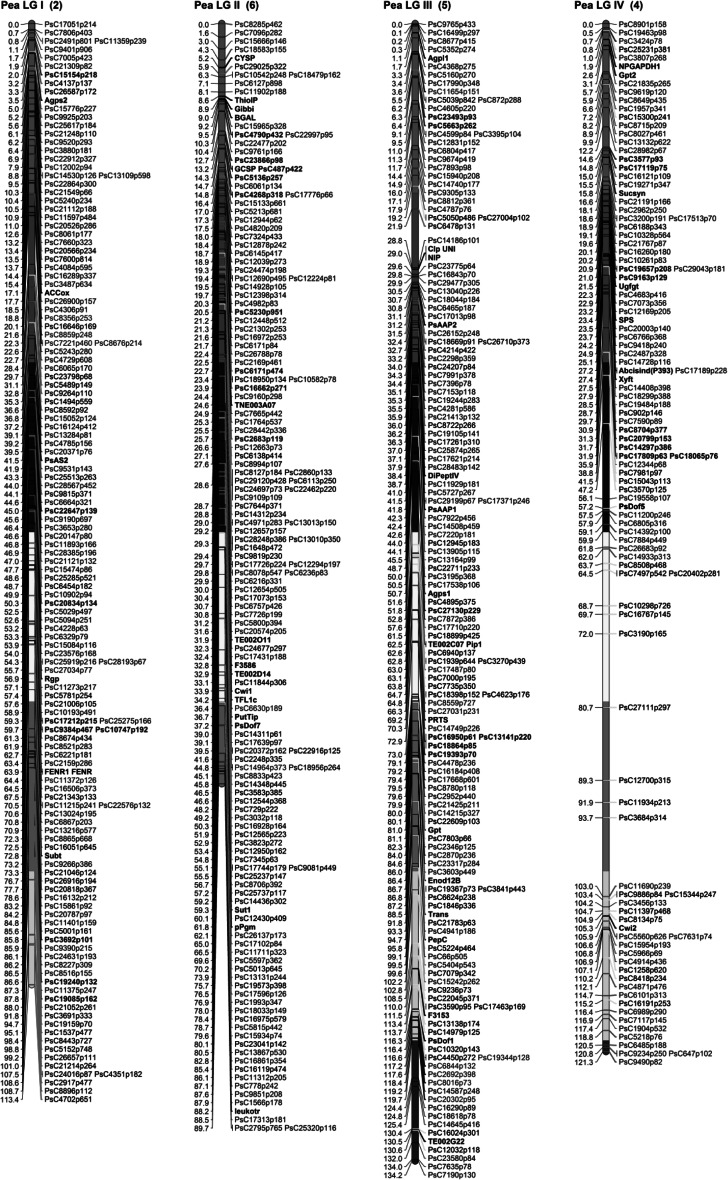

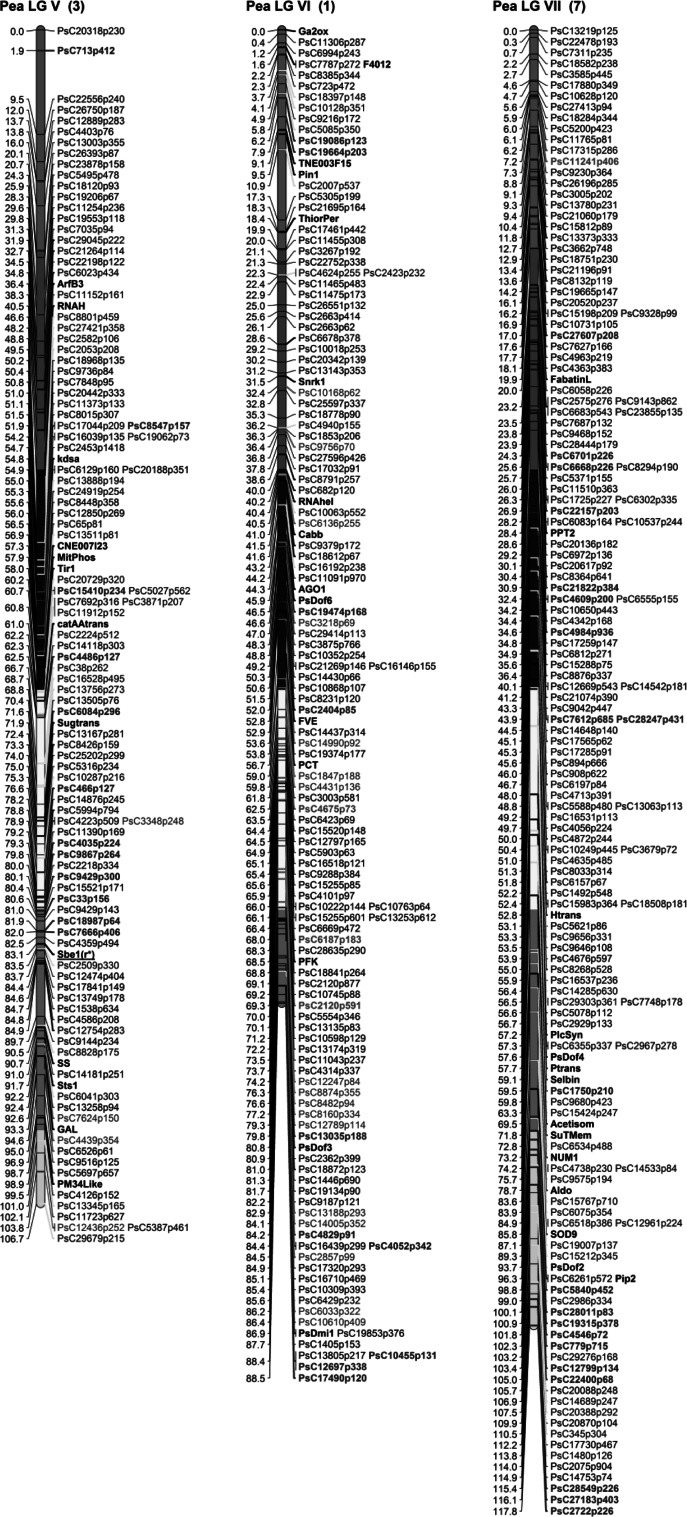
Consensus SNP linkage map of *Pisum sativum* generated by using five RIL mapping populations, with LG I–IV in (**a**) and LG V–VII in (**b**). The seven linkage groups (LG I–VII) representing 7 chromosomes (given in parenthesis). Anchor markers identifying the linkage groups are shown *black bold*. The SNP markers common to all five RILs are shown *bold red*. SNP markers unique to individual RILs PR-02 (*green*), PR-07 (*dark blue*), PR-15 (*brown*), PR-19 (*pink*) and Pop-9 (*light blue*) are shown. *Black* SNP markers represent those markers shared by two or more of the RIL populations. A total of 939 loci are represented in the consensus map. The division of linkage groups into 20 cM recombination bins is represented with blocks of *different colors*. *Asterisk* Mendel’s *r* (‘rugosus’ trait, where cotyledons are wrinkled with compound starch grains) is indicated on LG V (color figure online)

Individual linkage maps were constructed. Maximum likelihood mapping was carried out using using JoinMap 4.0 (Van Ooijen 2006), and a minimum LOD grouping threshold of 5. Regression mapping was used to finalize the map order of each linkage group. The Kosambi mapping function was used to convert the recombination frequencies into cM. The Pop9 map was constructed first and included 94 frame-work markers identified in earlier studies (Aubert et al. 2006; Deulvot et al. 2010). This allowed for the identification of individual linkage groups (LGs) and their orientation. The cross correspondence of Pop9 mapped SNP markers on individual maps was used to identify the equivalent LGs in all the RIL populations, and these were combined for map integration using the function ‘join groups’ in JoinMap 4.0. The consensus map output was used to generate a graphical map using MapChart 2.2 (Voorrips 2002). The consensus map was divided into 20 cM long recombination bins to better define the position of framework markers on the consensus map. The graphical color-coded consensus pea map showing the markers contributed from each population and bin distribution is presented in Fig. 1. The open source browser-based comparative analysis programme CMap (version 1.01; http://gmod.org/wiki/Cmap) was also utilized for the visualization of individual maps and to assess the congruency of marker positions and order among different genetic maps.

### Comparative analysis of the pea genetic map with *Medicago truncatula* and lentil

An assessment of the syntenic relationship between pea, *M. truncatula* and lentil was undertaken using the in silico mapping data of polymorphic pea contigs mapped using BLAT (Wu and Watanabe 2005) to orthologues in the model genome together with output from a similar analysis with lentil (Sharpe et al. 2013). The comparative mapping data of macrosyntenic blocks for both pea and lentil against *M. truncatula* were visualized using the Circos plotting tool application (Krzywinski et al. 2009; http://circos.ca/).

### Data availability

KnowPulse (http://knowpulse2.usask.ca/portal) is a repository of legume genetic and genomic data for pulse crop breeding. The assembled 454 sequencing data for the pea reference cultivar CDC Bronco is available with all polymorphic loci and associated markers (KASP and Illumina GoldenGate arrays) indicated. Potential homologues of a gene of interest can be identified using a BLAST interface based on sequence similarity. This allows for identification of existing markers associated with the gene of interest, as well as export information on specific loci in a variety of formats to aid in marker design. A comparative GBrowse [Generic Model Organism Database (GMOD), http://gmod.org] with a *Medicago* genomic backbone graphically displays sequence similarity-based homology between legume species providing an alternative approach to finding candidate markers. For an overview of all data made available through this project, visit the project page (http://knowpulse2.usask.ca/portal/node/3214575). All raw read data have been submitted to the NCBI-NIH Short Read Archive (BioProject ID: PRJNA237996; CDC Bronco (SRX555302); Alfetta (SRX555303); Nitouche SRX555304, Cooper (SRX555305), Striker (SRX555306), Orb (SRX555307), PI358610 (SRX555310) P651 (SRX555311)).

## Results

### 454 sequencing, SNP and SRR discovery

Across all eight pea accessions, 4,008,648 sequencing reads were processed ranging from 520,797 reads in CDC Bronco to 593,701 reads in Orb (Table 1). CDC Bronco was used to generate the reference de novo assembly, resulting in a set of 29,725 high-quality reference contigs representing a significant proportion of the 3′ end of genes in pea. Using the custom bioinformatics pipeline (Sharpe et al. 2013), the CDC Bronco reference assembly and the 3′-cDNA 454 sequence data from the seven *Pisum* accessions, a raw total of 131,425 SNPs were identified in 20,329 contigs (68 % of the total). Of these, 6,701 (30.6 %) non-redundant contigs were identified with significant homology to segments of the sequenced genomes of *Medicago* or soybean. Of this subset, 4,066 (60.7 %) had significant hits to annotated genes in *Medicago*, 1,048 (15.6 %) had significant hits to un-annotated regions in *Medicago*. Of those with no significant hit in *Medicago*, 1,587 (23.7 %) had a significant hit to annotated genes within soybean. These contigs collectively represent 20,008 SNPs (Table 1). Information relating to the orthologous genes in the model is important since it enables an inference of the genomic position of contigs within the *P. sativum* genome, and thus aids in the selection of an optimal set of evenly distributed SNP markers. The results of the in silico mapping of polymorphic contigs to *M. truncatula* homologues are provided in Supplementary Fig. 1.

Of the 20,008 SNPs, a set of 8,822 in 4,194 contigs could be utilized for GoldenGate SNP assay development based upon there being no other observable nucleotide variation flanking the target SNPs to compromise assay performance. This set of non-redundant and high-quality SNPs comprised of 5,667 transitions (64 %) and 3,079 transversions (36 %) with 76 SNPs that could not be classified due to ambiguity of bases in the reference. An average SNP frequency of 1 SNP per 667 bp in *P. sativum* cultivars was observed, while an average frequency of 1 SNP per 99 bp was observed when the *P. fulvum* and *P.* *sativum* spp. *abyssinicum* accessions were included. The average minor allele frequency for all SNP loci was 0.3 when all accessions were considered and 0.19 for the *P. sativum* cultivars alone. Five (16 %) of the 32 SNPs tested for validation failed across all genotypes (Supplementary Table S4). The other 27 SNPs were successfully separated by KASP assay, with 97 % successfully matching SNP calls with the 454 sequence data in each genotype. Three of the loci with errors were only incorrect in Nitouche and Orb, suggesting they could be heterozygous at these loci. Seven genotypes had no results in the 454 data, so represent new genotypic information for these cultivars. There were five instances of the KASP assays revealing the heterozygous nature of the cultivars, not seen in the 454 sequencing data which was derived from DNA extracted from a single plant. Details regarding the parameters for the selection of SNPs for the final pea GoldenGate array (Ps1536 OPA) are provided in the “Methods”.

An in silico analysis of a non-redundant set of 29,725 CDC Bronco contigs for the presence of microsatellite repeats revealed 406 (1.4 %) contigs that contained such a repeat with 64 (16 %) of these with a potential polymorphism based upon the available sequence data for all genotypes (Supplementary Table S5).

### SNP genotyping and genetic mapping

Five pea RIL populations (Table 2) were genotyped with the Ps1536 GoldenGate OPA. Based upon genotype scores in the parental lines alone, a wide range of polymorphic loci was identified between 340 (22 %; PR-02) and 940 (61 %; PR-19) loci (Table 2; Supplementary Table S3). Between 457 (30 %; PR-19) and 1,043 (68 %; PR-07) of the genotype calls were monomorphic in any one parental comparison and only a few dominant alleles were observed (ranging from 7 in PR-02 to 49 in PR-19). Six to nine percent of assays failed or had results that were unscorable in any one of the five populations, thus falling within Illumina specifications for expected success rate for new GoldenGate assays (90 % assay success). In total, 1,009 (66 %) SNPs out of 1,536 on the array were polymorphic in at least one RIL population and could be included in linkage analysis.

Single-nucleotide polymorphism loci exhibiting a distorted segregation with parental allele frequency of between 0.1 and 0.3 were identified in all populations but mainly in PR-19 (1 from PR-02, 1 from PR-07, 3 from PR-15, 90 from PR-19 and 9 from Pop9). However, the majority (90 %) of these distorted loci exhibited a balanced frequency greater than 0.3 in at least one of the other populations. All but 24 SNP markers from PR-19 with allele frequencies between 0.1 and 0.3 revealed a more balanced frequency in at least one of the other RIL populations. A total of 535 (57 %) loci identified as polymorphic in the parents of the PR-19 population (a cultivar × wild accession population) exhibited extreme segregation distortion with an allele frequency below 0.1 and these loci were not included for further analysis.

The pair-wise comparisons of SNP markers shared by RIL populations are summarized in Table 2. The number of shared SNPs ranged from 57 between PR-07 and PR-19 to 138 between PR-02 and Pop9. A total of 527 (34 %) of the SNP markers on the Ps1536 array did not produce segregation data suitable for linkage analysis in any of the five populations. Of these, 348 were polymorphic amongst parental lines but exhibited extreme distortion in different RIL populations, 134 were monomorphic across all populations, and 45 produced unscorable genotyping reactions or failed across all populations.

**Fig. 2:**
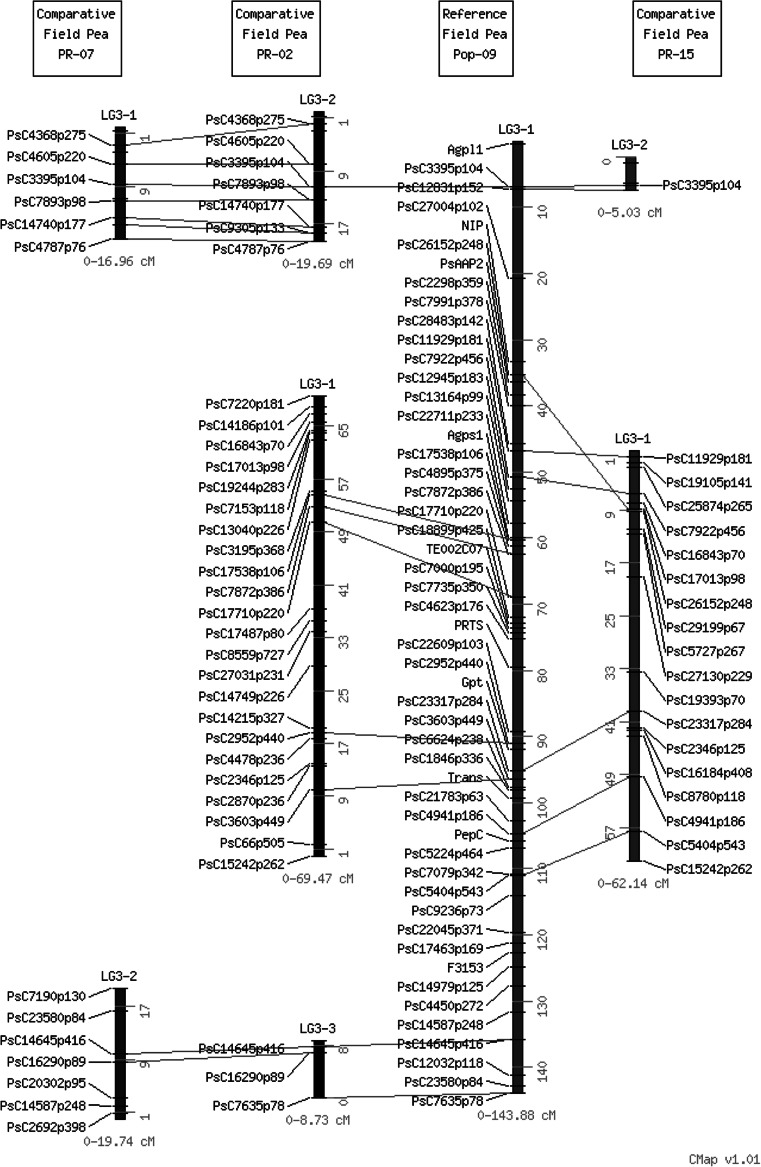
A comparative alignment of LG3 in four RIL populations (right to left; PR-07, PR-02, Pop-9 and PR-15 using CMap version 1.01. Common markers between groups are highlighted in *red* to visually represent synteny of marker orders and marker positions (color figure online)

The size of the individual maps of the five mapping populations ranged from 358.02 to 691.89 cM with the total of mapped markers between 323 (PR-02) and 492 (Pop9). The Pop9 map also had 94 framework markers and the maximum marker interval distance was 13.51 cM. The analysis allowed the placement of 939 of the 1,009 polymorphic SNP loci to unique positions on the seven linkage groups (PsLGs) of the consensus map covering 771.6 cM (Fig. 1). Each population contributed unique markers covering all seven LGs. The unique markers contributed by individual RIL populations to the final consensus map are color-coded in Fig. 1. While the consensus map condensed to the expected seven LGs, the individual maps ranged between 8 LG (PR-19) and 21 LGs (PR-15) (Supplementary Table S3). The consensus map was divided into 42 recombination bins, 20 cM long. Out of these, 9 bins did not have any framework marker, while 33 had at least one framework marker with 1 bin having a maximum of seven framework markers. The number of markers present in each bin ranged from 4 to 63 markers (Supplementary Table S6). To better assess and confirm the congruency of marker positions and order among different genetic maps, a comparative analysis using CMap was carried out for the four RILs derived from cultivated parents (Fig. 2; Supplementary Fig. 2). Although overall congruence and order was good, the possibility of rearrangements on some LGs relative to different RILs was identified, perhaps most clearly at the top of PsLGVI among Pop9, PR-02 and PR-07 RILs.

Examination of the contribution of loci to the consensus map from the individual RIL populations identified groups of loci that were unique or enriched in particular populations (Fig. 1). For example, 14 markers on the end of PsLGI were only polymorphic in PR-19 while similar groups of loci are also evident on PsLGII (two blocks in PR-19; 14 markers in one and 11 in a second block), the end of PsLGIV (13 markers in PR-02) and the middle of PsLGVII (10 markers in PR-07). The 13 clustered markers mapped to PsLGIV in PR-02 had been expected to be monomorphic based on the parental genotypes from the original 3′-cDNA 454 sequence data.

### Synteny between *P. sativum,M. truncatula* and *L. culinaris*

**Fig. 3:**
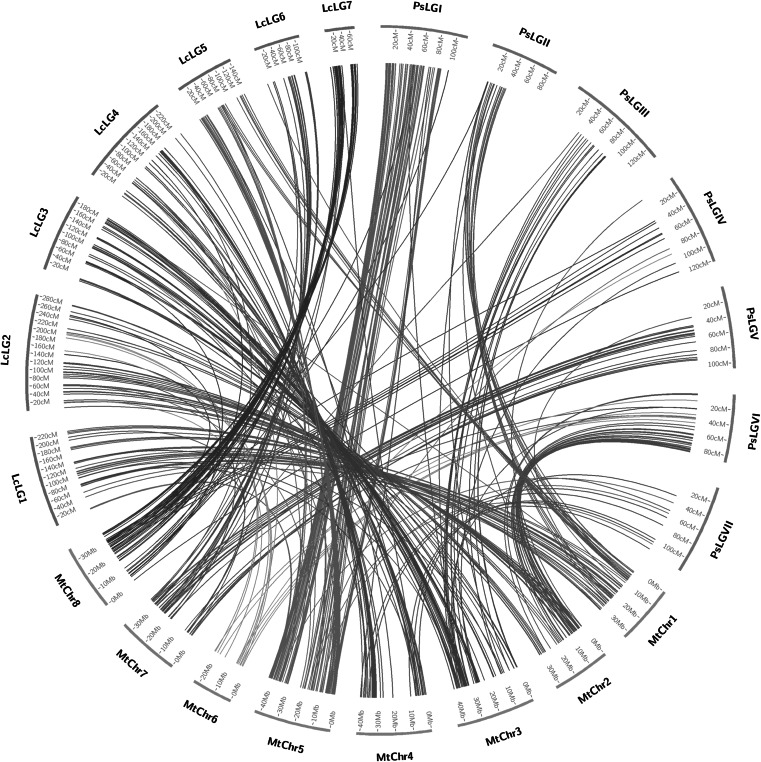
Syntenic relationships visualized by the Circos viewer (Krzywinski et al. 2009) showing extensive synteny of pea and lentil (Sharpe et al. 2013) linkage groups with the *M. truncatula* pseudochromosomes (Young et al. 2011). Ps = *Pisum sativum* (pea), Lc = *Lens culinaris* (lentil), and Mt = *Medicago truncatula*

Comparative mapping of pea relative to both *Medicago* and lentil revealed large segments of shared synteny with both species (Fig. 3; Supplementary Fig. 3). PsLGI, PsLGII, PsLGIII, PsLGIV, PsLGV, and PsLGVII were relatively collinear along their entire length with individual *Medicago* chromosomes 5, 1, 3, 8, 7, and 4, respectively (Table 3). Pea PsLGVI exhibited shared synteny with segments of both chromosomes 2 and 6, while no other pea LG exhibited strong homology with these two chromosomes. Based on the observed syntenic relationship between the lentil LGs and the *Medicago* chromosomes (Sharpe et al. 2013), it was possible to infer large regions of shared synteny between pea and lentil (Fig. 3; Supplementary Fig. 3; Table 3) although in some regions the synteny is more limited (Supplementary Fig. 3).

**Table 3:** Correspondence among pea linkage groups and *M. truncatula* pseudochromosomes and lentil linkage groups

*P. sativum*	*M. truncatula*	*L. culinaris*
I	5	5
II	1	1, 5
III	3	3
IV	8	7
V	7	6
VI	2, 6	2
VII	4	4

## Discussion

Genetic mapping is a pre-requisite for applications of marker-assisted selection and map-based cloning and significant efforts are being made in many crop species to develop saturated molecular maps. Despite significant advances made in the development and adoption of high-throughput genotyping and mapping tools, progress in the development of saturated molecular maps has been slow. The majority of genetic linkage maps now available have been developed by groups working independently on a wide range of mapping populations. Multiple linkage maps developed from different mapping populations recently have been aligned to generate consensus linkage maps by using common markers as framework markers. These markers identify linkage groups and define regions and orientation of the maps (Isobe et al. 2009; Gustafson et al. 2009). The use of multiple mapping populations has helped in identifying additional polymorphic markers (Studer et al. 2010; Gautami et al. 2012). In this study we used 94 previously published framework markers with known locations on linkage groups to generate a consensus linkage map from five RIL populations in pea. These markers are more or less evenly distributed over all the linkage groups with an average of 12 framework markers per linkage group. A minimum of three framework markers per linkage group is suggested to be sufficient for determining orientation of the map (Gautami et al. 2012). The development of consensus maps from independent maps generated by multiple mapping populations using JoinMap software may result in discrepancies in linear order of markers due to combining of data from mapping populations with different recombination frequencies, genetic background, size of population, and marker density (Feltus et al. 2006). For this reason comparative mapping using CMap software was conducted to more efficiently visualize marker order in four pea RILs; an approach that aided in assessing and confirming the overall congruency of marker positions and order but also hinting at the possibility of some rearrangements on some LGs.

In our study, the markers showed varied segregation distortion in different RIL populations with PR-19 exhibiting the highest segregation distortion. Such variation in segregation distortion in individual mapping populations and inconsistencies in linear marker order in individual population maps and consensus maps have been reported previously (Gautami et al. 2012). To address these issues, several studies have reported the use of framework markers with known positions to divide linkage groups into segments representing bins which hold defined locations on chromosomes. Introduction of bins in linkage groups have been used in many crop species where saturated linkage maps are still not available (Gardiner et al. 1993; Kleinhofs and Graner 2001; Studer et al. 2010; Gautami et al. 2012). These bins can then define a fixed location on chromosomes having a framework marker. In this study, we divided the pea linkage groups into 20 cM long bins. Out of 42 bins, the majority had an anchored framework marker identifying their fixed position on linkage groups. Introduction of bins with framework markers will help the addition of more markers in defined regions and further develop the consensus map.

The same custom strategy for SNP discovery using 454 sequencing technology as described for lentil by Sharpe et al. (2013) was deployed in this study for the identification of a large number of SNPs from a diverse set of six pea cultivars, a wild accession of *P. fulvum* and a *P.* *sativum* subspp. *abyssinicum* land race. The 3′-cDNA profiling approach employed provided a robust data set with deep coverage of the targeted 3′ ends of genes for each of the eight genotypes. The deep coverage is significant since it provides the ability to derive both a robust reference de novo assembly for a large proportion of expressed transcripts from the harvested tissues, as well as increasing the probability of identifying highly confident SNPs for each of the genotypes. Indeed it was possible to identify a total of 29,725 non-redundant reference contigs for the CDC Bronco cultivar. This number of contigs represents a significant proportion of the expressed genes based on the fact that the diploid pea genome likely has similar gene content to the closely related model *Medicago* genome (45,888 protein coding transcripts; www.phytozome.net). Unfortunately, it is not possible to establish if particular transcript contigs are associated with particular tissues since the methodology utilized did not provide an efficient format for sample indexing, however, it is possible, via comparative sequence analysis, to infer both the gene function and expression characteristics from the model legume genomes, such as *Medicago* (Young et al. 2011) or soybean (Schmutz et al. 2010).

The identification of SNPs from the different *P. sativum* and *P. fulvum* accessions also employed the same bioinformatics pipeline previously employed in lentil (Sharpe et al. 2013) and enabled the identification of a raw total of 131,424 SNPs across 20,328 (68 %) of the reference contigs. The level of nucleotide diversity within the *P. sativum* genotypes appears to follow the adapted origin of the genotypes where lower levels are observed between genotypes adapted to a North American temperate climate, and higher levels are observed between these genotypes and the *P. sativum* ssp. *abyssinicum* genotype (PI 358610) or the wild *P. fulvum* genotype (P651). The two wild accessions were included to broaden the genetic base of the germplasm being evaluated and thus to increase the chances for identifying SNPs. *Pisum sativum* subspp. *abyssinicum* is thought to have arisen in an independent domestication from *Pisum sativum*, as has *Pisum fulvum*. These initial SNPs were filtered to a smaller high-quality set of SNPs based upon read coverage/depth and low levels of ambiguity (<3 reads or <80 % concordance), as well as identified significant homology to either the model *Medicago* or soybean genomes. This identified a set of 20,008 high-quality SNPs across 6,707 annotated contigs. The significant decrease in the number of high-quality SNPs and contigs likely indicates two things; (1) even though the methodology enables targeted profiling of discrete regions of genes, its effect is diluted because of the presence of a large number of transcripts from a relatively small number of genes in any given tissue type; and (2) the 3′ end of genes can contain large stretches of untranslated regions (UTR) that can have poor levels of sequence homology even with quite closely related species. The set of SNPs selected for SNP assay development (8,822 SNPs in 4,194 contigs) exhibited ratios of transition SNPs (64 %) to transversion SNPs (36 %) and a SNP frequency in *P. sativum* cultivars (1 SNP per 667 bp) that closely reflects the types of nucleotide conversions and frequency seen in similar transcript studies in pea using 454 sequencing (Kaur et al. 2012; Leonforte et al. 2013), other pulse crops such as lentil (Sharpe et al. 2013), as well as other plants (Soltis and Soltis 1998). A small number (406 (1.6 %)) of the CDC Bronco reference contigs were also found to contain a range of different microsatellite repeats, and an in silico analysis of the equivalent repeats in the sequence data from the other genotypes identified putatively polymorphic repeats. These microsatellite loci represent a resource for potential genetic marker development and complement identified repeats from other initiatives using similar sequencing strategies in this crop (Loridon et al. 2005; Kaur et al. 2012).

To select an optimal set of SNPs for representation on the Illumina GoldenGate 1,536 SNP array, the 8,822 high-quality SNPs identified were screened for the removal of those SNPs that were potentially heterozygous in nature based upon the available sequence data. A total of 1,018 SNPs were identified as such indicating the residual heterozygosity in the genotypes that were selected for SNP discovery. This level of heterozygosity is not unexpected given the nature of the breeding approaches used to develop the pea cultivars in this research, i.e., none were derived from doubled haploidy. These SNPs, while potentially useful polymorphisms, were not selected for SNP assay development because we could not be confident they would be present in the targeted RILs. Of the remaining SNPs that were submitted to Illumina for assay design, it was possible to identify a set of 3,106 SNPs that best represent single contigs. This was based upon selecting SNPs with highest ADT score in cases where multiple SNPs were present in one contig. This strategy performed very well when implemented for the design of an equivalent GoldenGate 1,536 SNP array in lentil (Sharpe et al. 2013). As was the case for the lentil array, further filtering of the SNPs based upon the presence of variation amongst only the cultivated lines, the removal of lower ADT assay scores (<0.4) and the removal of SNPs only identified in a limited number of lines due to limited amounts of available sequence data enabled the selection of a core set of optimal SNPs to be represented on the array (1,107 SNPs). It should be noted that one difference from the lentil effort described by Sharpe et al. (2013), was that a more robust methodology for the identification of identical positional SNPs in duplicate overlapping contigs was employed in this effort; multiple approaches to map either contigs or individual reads to reference *Medicago* gene models were undertaken using both BLAT and GMAP. This was carried out in an effort to avoid the possibility of designed assays amplifying multiple loci within the pea genome. The supplementation of the selected 1,107 SNPs for representation on the array with an additional set of 429 markers only polymorphic between the *P. fulvum* and *P. sativum* ssp. *abyssinicum* genotypes and CDC Bronco completed the 1,536 SNP array. The representation of these SNPs on the array provides an enhanced ability not only to assess diversity across a wider range of germplasm, but also to better characterize segregating progeny from *P. sativum* × *P. fulvum* and *P. sativum* × *P. sativum* ssp. *abyssinicum* crosses that are available in pea breeding programs.

Validation of the selected 1,536 SNPs was established by using a subset of 32 markers for the design of KASP markers and screening against genotypes used for SNP discovery. From this it was possible to confirm that the majority of the assays would perform as expected in the larger format. A subset of five markers (16 %) did not amplify at all, and although an amount of assay failure is to be expected a subsequent analysis of these SNPs indicated that one of them had a flanking SNP in the assay design space and two of them were closely adjacent to exon/intron boundaries within the orthologous *Medicago* or soybean gene models. Variation in such boundaries between closely related species is to be expected and is a limitation of an approach where transcriptome data from a genome without an established reference genome is being used for SNP discovery and marker development. The validation exercise also confirmed that a significant amount of heterogeneity exists within established cultivars since it was revealed that several markers detected alleles in the cultivars that were different from the observed polymorphism in the sequence data used for SNP discovery.

The utilization of the Ps1536 GoldenGate array for genotyping in the five pea RIL populations was successful in identifying subsets of polymorphic SNP assays within each of the populations. The large difference in levels of polymorphism among the RIL populations is to be expected based upon the diverse nature of the parental material used to develop the populations. As expected the most polymorphic population (61 % polymorphic loci) was PR-19, derived from a cross between the *P. sativum* cultivar Alfetta and the wild *P. fulvum* accession P651. The remaining populations each had between 22 % (PR-02) and 26 % (Pop9) polymorphic loci, the former being derived from a cross between two modern cultivars (Orb and CDC Striker) and the latter between a modern cultivar and a Chinese landrace (Cameor and China). The substantially greater number of polymorphic loci in the PR-19 cross likely reflects the very large degree of nucleotide diversity that exists in genic regions between the two species (Jing et al. 2007), while the level of polymorphism observed in Pop9 reflects the more moderate levels of nucleotide diversity that exists within *P. sativum*, even when including landraces. A small number of failed or unscorable assays (6–9 %) in each population is expected based upon the observed failure rate for the GoldenGate 1,536 SNP assay format (Cunningham et al. 2008), as well as the limitation of the design process inferring exon/intron boundaries from a related model species reference genome. The small number of assays where only one parental allele was observed (i.e. dominant loci) was very small with only Pop9 and PR-19 populations revealing any significant number (approx. 3 % each), again likely reflecting the quite diverse nature of these crosses.

The 1,009 (66 %) SNP assays that produced segregation data suitable for linkage analysis reflected the desire to design an array that would provide utility for genetic mapping across a broad range of crosses. The bi-allelic nature of the SNP assay format means that in any one *P. sativum* cross a relatively small proportion of loci (22–26 %) are polymorphic, but collectively a much larger proportion of loci can be mapped. In theory a much higher number of loci could have been mapped in PR-19 since 940 loci were identified as polymorphic between the parental lines, however, many of these loci (57 %) exhibited extreme segregation distortion (allele frequency <0.1) that was unique to this population. Of note, the majority of these loci revealed distortion skewed toward the cultivated parent genotype. The scale of this distortion is such that it indicates there were serious factors influencing the expected Mendelian ratios in the RIL population. It is possible that significant amounts of heterozygosity existed in the *P. fulvum* parent of the F_1_ such that an alternative undetectable allele is present at many loci and which resulted in the levels of distortion we observed. It is also possible that multiple gametophytic and/or genetic factors limited the representation of one parental allele over the other allele in a segregating population and there are many such reported examples in different crops (for review see Liu et al. 2010). In this case multiple significant genetic differences, such as large chromosomal translocations and inversions that are known to exist between *P. sativum* and *P. fulvum* (Errico et al. 1991), may have caused abnormal chromosome segregation in the F_1_ used to generate the population. Linkage analysis of the segregation data in the five populations produced individual genetic maps for each cross that had significantly different sizes. The size of 345.3 cM for PR-19 is particularly small and again suggests that although the parents are very polymorphic only a portion of the genome is segregating normally in this interspecific cross.

All of the maps individually contained eight or more independent linkage groups, following the same observation in a single population described by Leonforte et al. (2013) and indicating the limitations of achieving complete genome coverage in any one bi-parental cross with a limited number of polymorphic loci. The consensus genetic map, however, derived from the five data sets, contained a total of 939 mapped loci across seven LGs with a total genetic distance of 771.6 cM for the genome. The utilization of framework SSR markers enabled a robust integration of the consensus map with PsLG I-VII in the Pop9 map generated previously (Bordat et al. 2011). The ability to identify groups of loci within the consensus map that were primarily derived from particular crosses also established that certain regions of the genome were only polymorphic in particular crosses, most notably PR-19 and Pop9. This is not surprising considering the diverse nature of these crosses. The significant number of the groups of loci contributed by the interspecific cross PR-19 indicates that even though the cross may have a significant issue relating to normal chromosome segregation it was still possible to use the data to enhance the breadth of the consensus genetic map. The identification of clusters of loci on LG II (between marker Gibbi and cwi1), LG III (between markers PsAAP1 and NIP), and LG IV (between markers Sucsyn and Xyft) with moderate segregation distortion in individual RIL populations was also evident, with the region on LGII confirming the previously identified distortion in the region between anchor markers Gibbi and Cwi1 on this group in Pop9 (Bordat et al. 2011).

The benefit of developing these resources in pea using transcriptome data is that it is possible to take advantage of strong sequence similarity in genic regions between pea and the available sequence data from the closely related model *Medicago* genome (Young et al. 2011). Both pea and *Medicago* reside in the same galegoid clade within the papilionoid legume sub-family and are estimated to have diverged approximately 20 MYA (Cannon et al. 2009). This close relationship between the two species therefore offers the possibility of a detailed examination of the shared synteny that exists between their genomes. The availability of a similar transcriptome resource for lentil (Sharpe et al. 2013), which is another close relative within the galegoid clade, also enables a comparative analysis of synteny with both pea and *Medicago*.

This study identified very similar correspondence with respect to pea PsLGs, lentil LGs and *Medicago* chromosomes as those reported previously (Bordat et al. 2011; Smýkal et al. 2012; Leonforte et al. 2013; Sharpe et al. 2013), and previously observed rearrangements were also largely confirmed. For example PsLGI is largely collinear with Mt5, except for a small inversion of markers in the centre of the group and half of this chromosome reveals good synteny with lentil LG5. Similarly, PsLGII is largely syntenic with Mt1 with a large inversion at one end of the group while this chromosome has synteny with both lentil LG1 and LG5. Likewise PsLGIV has shared synteny with Mt8 which in turn has substantial shared synteny with lentil LG7. Interestingly, significant shared synteny between PsLGIII and Mt3 could be established with no evidence for shared synteny with Mt2 as described by Bordat et al. (2011). This lack of shared synteny could indicate technical limitations with the nature of the 3′ transcript profiling and resultant short contigs consisting of 3′ UTR sequences with little homology to the *Medicago* gene models, or the possibility of erroneous linkages being established between markers in the maps. It is also possible that regions of the pea genome could be more distinct because of large scale genome reorganization since the two species diverged or potentially there are specific chromosome translocations within different pea cultivars. Genome reorganization since divergence could also explain the situation in lentil where only half of Mt5 revealed substantial synteny with lentil LG5, whereas the whole chromosome is fully syntenous with PsLGI.

The genetic and genomics resources described here hold the promise to accelerate on-going efforts to improve *P. sativum* productivity and seed quality by providing a mechanism to manipulate useful variation in the crop and to analyze complex polygenic traits (e.g. QTL analysis). Together with other resources that have been recently developed (e.g., Leonforte et al. 2013), they will also assist in future efforts to develop a high-quality genome sequence for pea.

### **Author contributions**

AGS, KEB, BT, and TDW were the co-PIs on the projects that led to this manuscript; they conceived of the study, participated in its design and coordination and co-wrote the manuscript together with AS. AGS oversaw the 3′ cDNA library construction and sequencing portions of the work. AS, MD, RS, and KEB oversaw the genotyping and mapping portions of the work. TDW selected the germplasm used for sequencing and developed the PR-02, PR-07, PR-15, and PR-19 mapping populations together with BT, YL, ASKS, and ABJ, respectively. JB and GA developed the Pop9 mapping population. LAS prepared the data for dissemination through KnowPulse and provided bioinformatic support throughout the project. RL and JC provided input in terms of RNA extraction and established the adapted form of the 3′ cDNA profiling methodology. LR provided bioinformatics support from SNP selection through data analysis and prepared several figures. All authors read and approved the final manuscript.

## Electronic supplementary material

Below is the link to the electronic supplementary material.

Supplementary material 1 (DOCX 117 kb)

Supplementary material 2 (DOCX 153 kb)

Supplementary material 3 (DOCX 716 kb)

Supplementary material 4 (DOCX 16 kb)

Supplementary material 5 (XLSX 436 kb)

Supplementary material 6 (XLSX 9 kb)

Supplementary material 7 (DOCX 28 kb)

Supplementary material 8 (XLSX 848 kb)

Supplementary material 9 (XLSX 46 kb)
