# Supplementary material for: Gene-based SNP discovery and genetic mapping in pea
Source: Theor Appl Genet. 2014 Aug 15;127(10):2225–41. doi: 10.1007/s00122-014-2375-y (PMC4180032; doi:10.1007/s00122-014-2375-y)
Supplement: Supplementary file 1 — Supplementary material 1 (DOCX 117 kb) [file 122_2014_2375_MOESM1_ESM.docx]

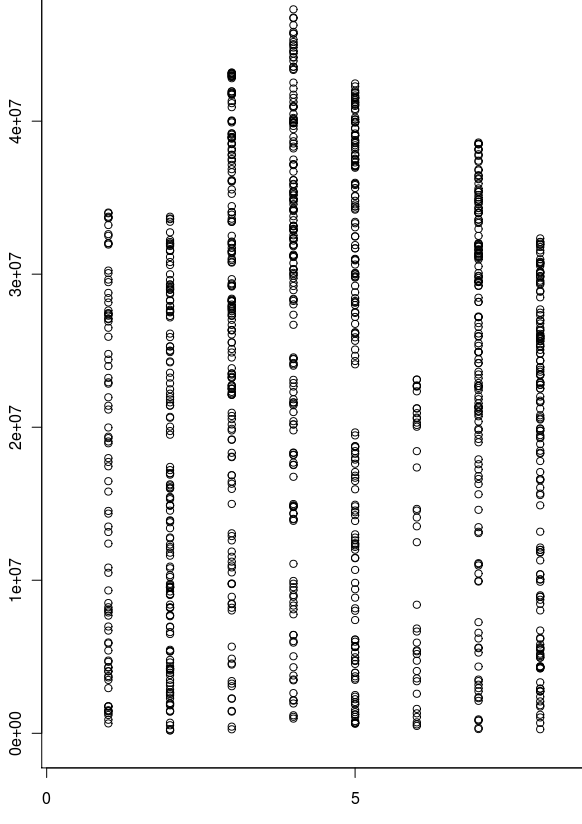


**Supplementary Fig. 1.** Schematic representation of the genome distribution of the 1,536 pea SNP loci selected for Illumina GoldenGate array development as inferred from the position (y axis: length in bp) across the eight *M. truncatula* pseudochromosomes (x-axis; Chr 1-8) (Young et al. 2011).
