# Supplementary material for: Gene-based SNP discovery and genetic mapping in pea
Source: Theor Appl Genet. 2014 Aug 15;127(10):2225–41. doi: 10.1007/s00122-014-2375-y (PMC4180032; doi:10.1007/s00122-014-2375-y)
Supplement: Supplementary file 2 — Supplementary material 2 (DOCX 153 kb) [file 122_2014_2375_MOESM2_ESM.docx]

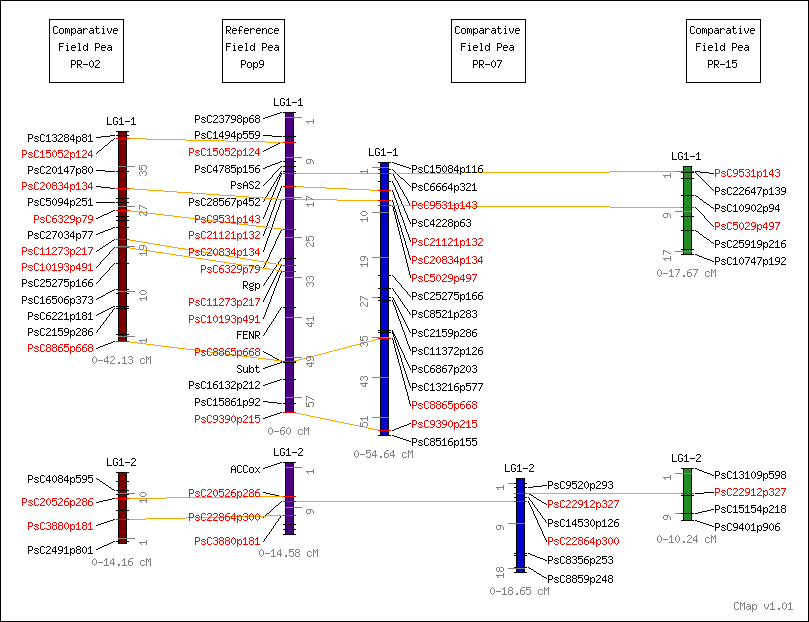


**Supplementary Fig. 2.** A comparative alignment of LG I in four RIL populations (right to left; PR-02, Pop-9, PR-07, and PR-15) using CMap version 1.01. Common markers between groups are highlighted in red to visually represent synteny of marker orders and marker positions.


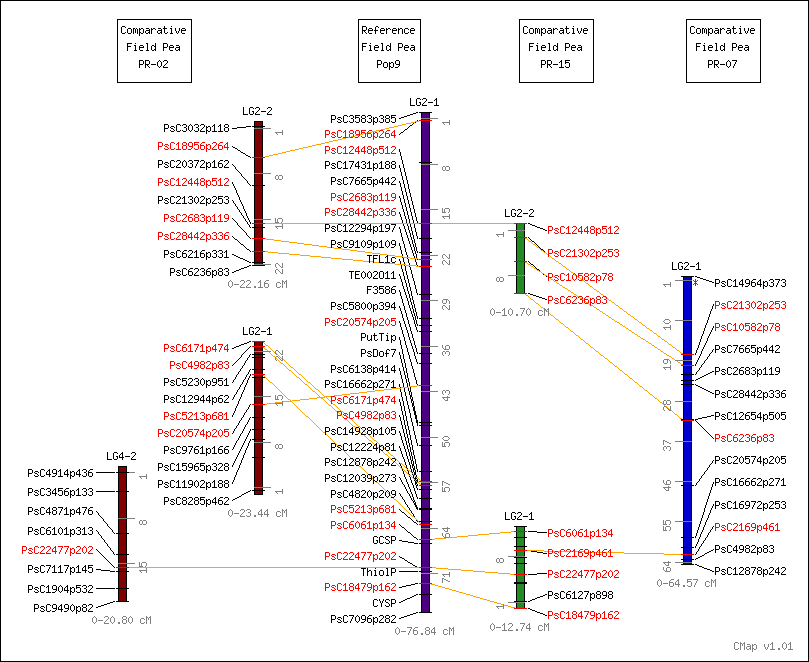


**Supplementary Fig. 2 cont.** A comparative alignment of LG II in four RIL populations (right to left; PR-02, Pop-9, PR-15, and PR-07) using CMap version 1.01. Common markers between groups are highlighted in red to visually represent synteny of marker orders and marker positions.


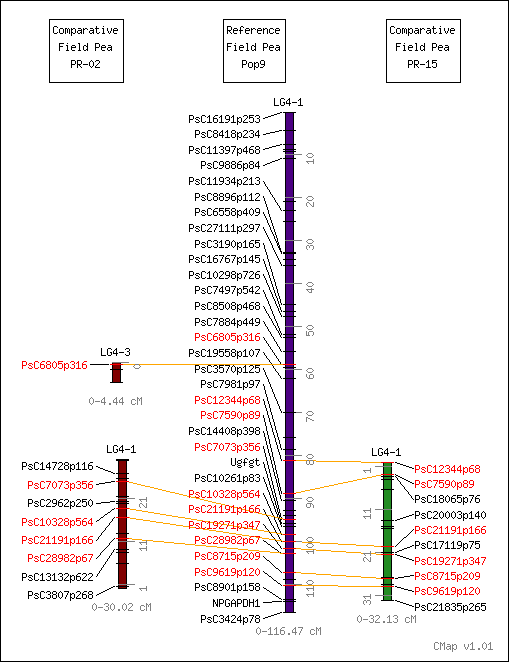


**Supplementary Fig. 2 cont.** A comparative alignment of LG IV in three RIL populations (right to left; PR-02, Pop-9, PR-15) using CMap version 1.01. Common markers between groups are highlighted in red to visually represent synteny of marker orders and marker positions.


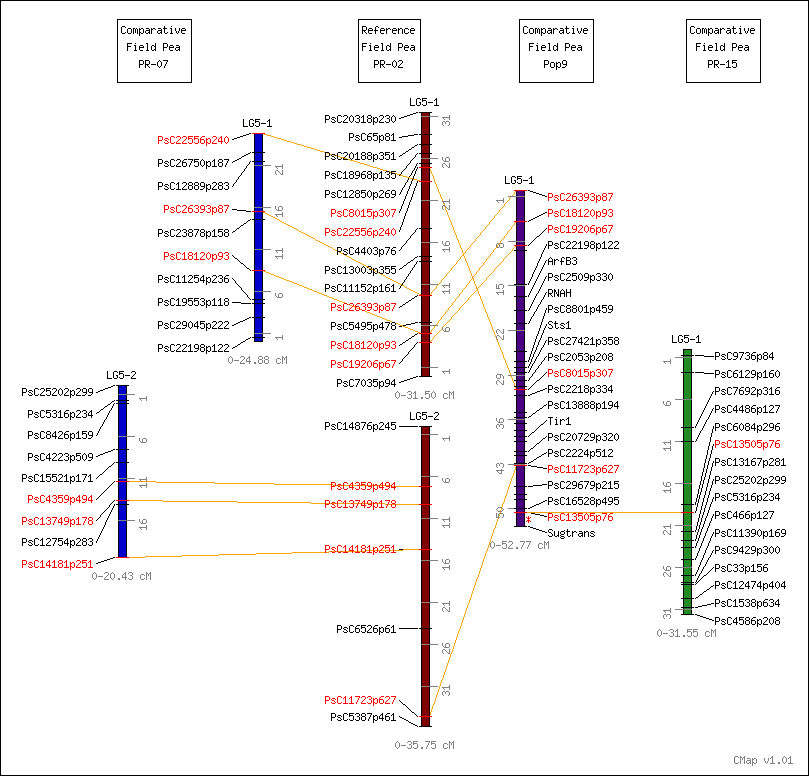


**Supplementary Fig. 2 cont.** A comparative alignment of LG V in four RIL populations (right to left; PR-07, PR-02, Pop-9, and PR-15) using CMap version 1.01. Common markers between groups are highlighted in red to visually represent synteny of marker orders and marker positions.


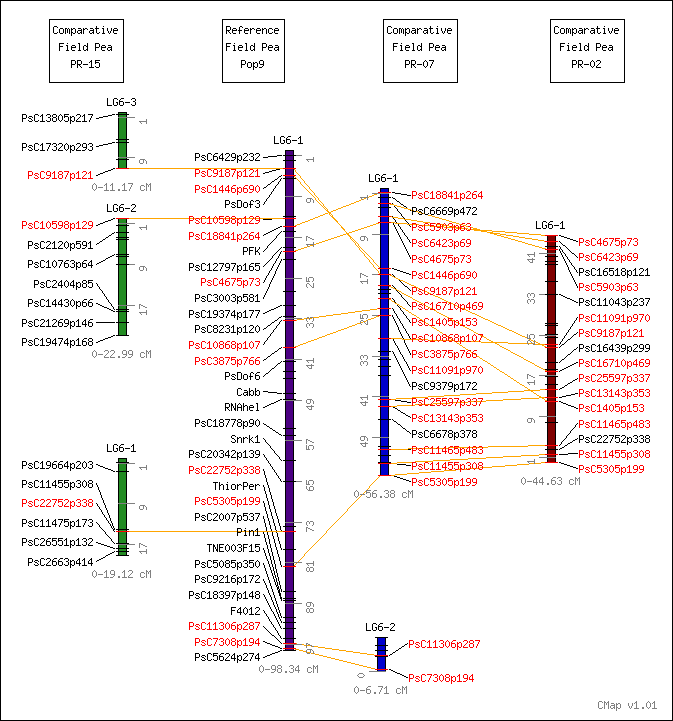


**Supplementary Fig. 2 cont.** A comparative alignment of LG VI in four RIL populations (right to left; PR-15, Pop-9, PR-07 and PR-02) using CMap version 1.01. Common markers between groups are highlighted in red to visually represent synteny of marker orders and marker positions.


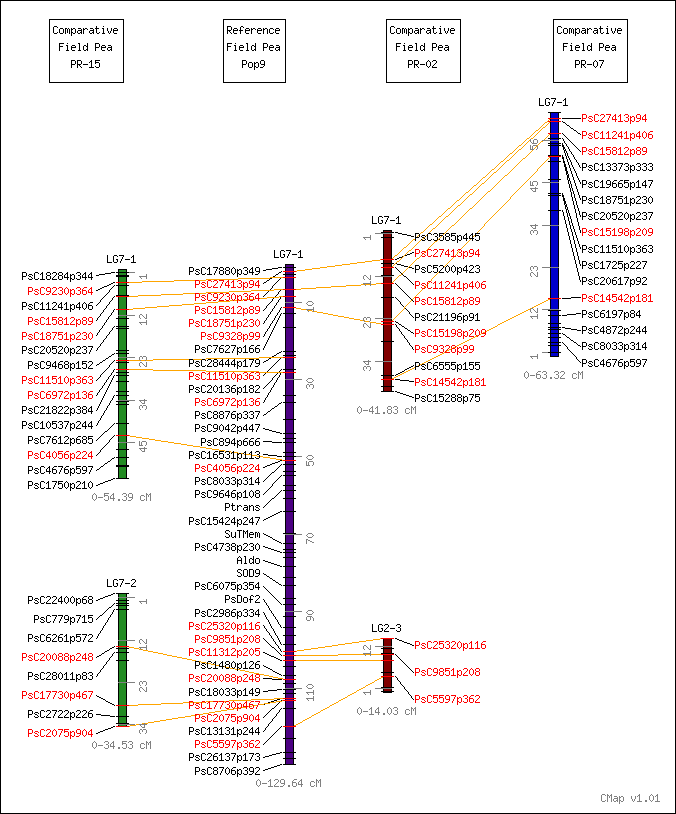


**Supplementary Fig. 2 cont.** A comparative alignment of LG VII in four RIL populations (right to left; PR-15, Pop-9, PR-02 and PR-07) using CMap version 1.01. Common markers between groups are highlighted in red to visually represent synteny of marker orders and marker positions.
