# Supplementary material for: Gene-based SNP discovery and genetic mapping in pea
Source: Theor Appl Genet. 2014 Aug 15;127(10):2225–41. doi: 10.1007/s00122-014-2375-y (PMC4180032; doi:10.1007/s00122-014-2375-y)
Supplement: Supplementary file 3 — Supplementary material 3 (DOCX 716 kb) [file 122_2014_2375_MOESM3_ESM.docx]

**
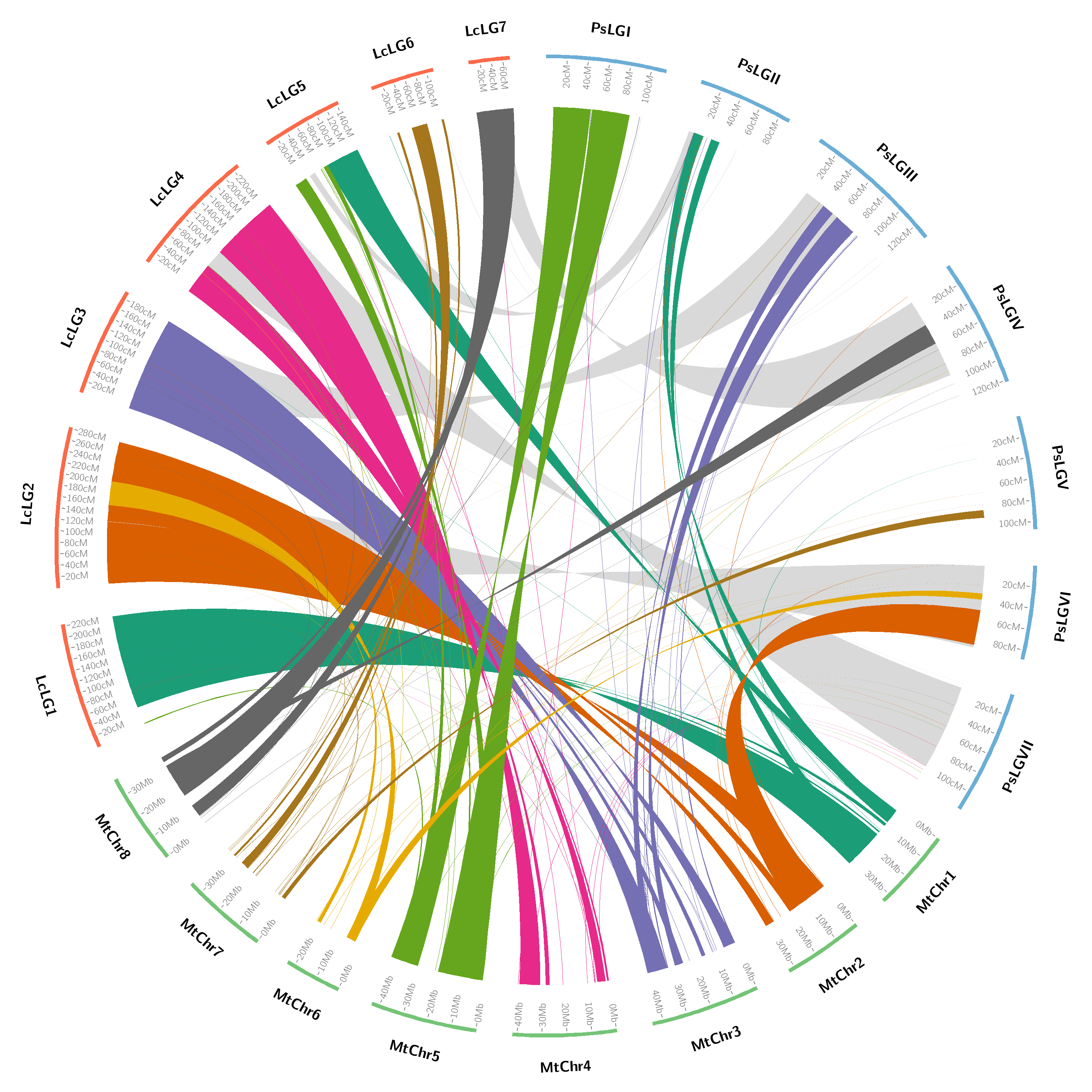
**

**Supplementary Fig. 3.** Syntenic relationships visualized by the Circos viewer (Krzywinski, M. et al. 2009) showing extensive synteny of pea and lentil (Sharpe et al. 2013) linkage groups with the *M. truncatula* pseudochromosomes (Young et al. 2011) (solid ribbon format) together with a representation of equivalent syntenic blocks between pea and lentil (grey shading).
