# Supplementary material for: Gene-based SNP discovery and genetic mapping in pea
Source: Theor Appl Genet. 2014 Aug 15;127(10):2225–41. doi: 10.1007/s00122-014-2375-y (PMC4180032; doi:10.1007/s00122-014-2375-y)
Supplement: Supplementary file 4 — Supplementary material 4 (DOCX 16 kb) [file 122_2014_2375_MOESM4_ESM.docx]

**Supplementary Table S1. Summary of origin of pea RIL populations**

| **Population** | **Female Parent and origin** | **Male parent and origin** | **Relevant References** |
| --- | --- | --- | --- |
| **PR-02** | Orb  (Sharpes, UK) | CDC Striker  (University of Saskatchewan, Canada) | Warkentin et al. 2004 |
| **PR-07** | Carrera  (Limagrain, Netherlands) | CDC Striker  (University of Saskatchewan, Canada) | Warkentin et al. 2004 |
| **PR-15** | 1-2347-144 (low phytate line derived from CDC Bronco (University of Saskatchewan, Canada) | CDC Meadow  (University of Saskatchewan, Canada) | Warkentin et al. 2005; Warkentin et al. 2007; Warkentin et al. 2012 |
| **PR-19** | Alfetta  (Limagrain, Netherlands) | P651 (*P. fulvum*)  (Spain  Instituto de Investigacion y Formacion  Agraria y Pesquera, Spain) | Fondevilla et al. 2005; Jha et al. 2012 |
| **Pop9** | Cameor  France | JI 1491 (Chinese accession, China) | Bordat et al. 2011 |
