# Supplementary material for: Gene-based SNP discovery and genetic mapping in pea
Source: Theor Appl Genet. 2014 Aug 15;127(10):2225–41. doi: 10.1007/s00122-014-2375-y (PMC4180032; doi:10.1007/s00122-014-2375-y)
Supplement: Supplementary file 7 — Supplementary material 7 (DOCX 28 kb) [file 122_2014_2375_MOESM7_ESM.docx]

**Supplementary Table S4. Summary of pea KASP validation assays**

| **KASP Assay Mix** | **Pea SNP** |  | **CDC Bronco** | **Alfetta** | **CDC Striker** | **CDC Cooper** | **Nitouche** | **Orb** | **Marker score** |
| --- | --- | --- | --- | --- | --- | --- | --- | --- | --- |
| **1** | **PsC1006p1115** | **SEQ** | **T** | **C** | **T** | **C** | **T** | **C** | **Perfect** |
|  |  | **KASP** | **T** | **C** | **T** | **C** | **T** | **C** |  |
| **2** | **PsC10188p73** | **SEQ** | **T** | **T** | **T** | **T** | **T** | **T** | **Perfect** |
|  |  | **KASP** | **T** | **T** | **T** | **T** | **T** | **T** |  |
| **3** | **PsC10193p461** | **SEQ** | **T** | **C** | **C** | **T** | **C** | **C** | **Perfect** |
|  |  | **KASP** | **T** | **C** | **C** | **T** | **C** | **C** |  |
| **4** | **PsC10249p445** | **SEQ** | **C** | **C** | **A** | **C** | **X** | **A** | **Perfect** |
|  |  | **KASP** | **C** | **C** | **A** | **C** | **C** | **A** |  |
| **5** | **PsC10287p216** | **SEQ** | **A** | **X** | **A** | **G** | **G** | **A** | **Perfect** |
|  |  | **KASP** | **A** | **A** | **A** | **G** | **G** | **A** |  |
| **6** | **PsC10298p726** | **SEQ** | **C** | **T** | **C** | **X** | **T** | **T** | **Perfect** |
|  |  | **KASP** | **C** | **CT** | **C** | **T** | **T** | **CT** |  |
| **7** | **PsC10309p393** | **SEQ** | **A** | **A** | **A** | **G** | **A** | **A** | **Good** |
|  |  | **KASP** | **A** | **A** | **A** | **G** | **A** | **G** |  |
| **8** | **PsC10350p165** | **SEQ** | **A** | **G** | **G** | **G** | **G** | **G** | **Perfect** |
|  |  | **KASP** | **A** | **G** | **G** | **G** | **G** | **G** |  |
| **9** | **PsC10364p259** | **SEQ** | **T** | **C** | **T** | **C** | **C** | **T** | **Perfect** |
|  |  | **KASP** | **T** | **C** | **T** | **C** | **C** | **T** |  |
| **10** | **PsC10455p131** | **SEQ** | **C** | **T** | **C** | **C** | **T** | **C** | **Perfect** |
|  |  | **KASP** | **C** | **T** | **C** | **C** | **T** | **C** |  |
| **11** | **PsC10465p232** | **SEQ** | **T** | **C** | **T** | **T** | **T** | **T** | **Poor** |
|  |  | **KASP** | **T** | **C** | **T** | **T** | **C** | **C** |  |
| **12** | **PsC1048p792** | **SEQ** | **G** | **G** | **G** | **T** | **G** | **G** | **Fail** |
|  |  | **KASP** | **U** | **U** | **U** | **U** | **U** | **U** |  |
| **13** | **PsC10495p653** | **SEQ** | **A** | **A** | **G** | **G** | **A** | **G** | **Perfect** |
|  |  | **KASP** | **A** | **A** | **G** | **G** | **A** | **G** |  |
| **14** | **PsC10583p147** | **SEQ** | **A** | **A** | **X** | **G** | **G** | **G** | **Perfect** |
|  |  | **KASP** | **A** | **A** | **G** | **G** | **G** | **G** |  |
| **15** | **PsC10624p612** | **SEQ** | **G** | **A** | **G** | **A** | **G** | **A** | **Perfect** |
|  |  | **KASP** | **G** | **A** | **G** | **A** | **G** | **A** |  |
| **16** | **PsC10650p443** | **SEQ** | **C** | **T** | **T** | **T** | **T** | **C** | **Perfect** |
|  |  | **KASP** | **C** | **T** | **T** | **T** | **T** | **C** |  |
| **17** | **PsC10754p437** | **SEQ** | **C** | **C** | **C** | **C** | **C** | **T** | **Perfect** |
|  |  | **KASP** | **C** | **C** | **C** | **C** | **C** | **T** |  |
| **18** | **PsC10937p188** | **SEQ** | **C** | **C** | **C** | **T** | **X** | **T** | **Good** |
|  |  | **KASP** | **C** | **CT** | **C** | **T** | **T** | **T** |  |
| **19** | **PsC11427p250** | **SEQ** | **T** | **A** | **A** | **T** | **T** | **A** | **Fail** |
|  |  | **KASP** | **U** | **U** | **U** | **U** | **U** | **U** |  |
| **20** | **PsC11465p283** | **SEQ** | **T** | **C** | **T** | **C** | **T** | **T** | **Fail** |
|  |  | **KASP** | **X** | **X** | **X** | **X** | **X** | **X** |  |
| **21** | **PsC11844p306** | **SEQ** | **A** | **A** | **A** | **T** | **A** | **A** | **Perfect** |
|  |  | **KASP** | **A** | **A** | **A** | **T** | **A** | **A** |  |
| **22** | **PsC11885p256** | **SEQ** | **G** | **A** | **A** | **G** | **G** | **A** | **Fail** |
|  |  | **KASP** | **U** | **U** | **U** | **U** | **U** | **U** |  |
| **23** | **PsC1207p102** | **SEQ** | **T** | **T** | **T** | **X** | **A** | **T** | **Perfect** |
|  |  | **KASP** | **T** | **T** | **T** | **T** | **A** | **T** |  |
| **24** | **PsC1258p473** | **SEQ** | **A** | **A** | **T** | **T** | **A** | **T** | **Perfect** |
|  |  | **KASP** | **A** | **A** | **T** | **T** | **A** | **T** |  |
| **25** | **PsC12831p152** | **SEQ** | **T** | **C** | **C** | **C** | **T** | **C** | **Perfect** |
|  |  | **KASP** | **T** | **C** | **C** | **C** | **T** | **C** |  |
| **26** | **PsC12889p283** | **SEQ** | **A** | **C** | **A** | **C** | **C** | **A** | **Good** |
|  |  | **KASP** | **A** | **AC** | **A** | **C** | **C** | **A** |  |
| **27** | **PsC12940p109** | **SEQ** | **A** | **G** | **G** | **G** | **X** | **G** | **Poor** |
|  |  | **KASP** | **G** | **G** | **G** | **A** | **G** | **G** |  |
| **28** | **PsC12944p62** | **SEQ** | **A** | **G** | **G** | **G** | **A** | **A** | **Poor** |
|  |  | **KASP** | **A** | **G** | **G** | **G** | **G** | **G** |  |
| **29** | **PsC12p441** | **SEQ** | **A** | **A** | **A** | **A** | **G** | **A** | **Perfect** |
|  |  | **KASP** | **A** | **A** | **A** | **A** | **G** | **A** |  |
| **30** | **PsC13219p125** | **SEQ** | **G** | **A** | **A** | **G** | **G** | **G** | **Good** |
|  |  | **KASP** | **AG** | **A** | **A** | **G** | **G** | **G** |  |
| **31** | **PsC13222p347** | **SEQ** | **T** | **C** | **T** | **C** | **C** | **T** | **Perfect** |
|  |  | **KASP** | **T** | **C** | **T** | **C** | **C** | **T** |  |
| **32** | **PsC13238p195** | **SEQ** | **T** | **T** | **C** | **C** | **C** | **T** | **Fail** |
|  |  | **KASP** | **X** | **X** | **X** | **X** | **X** | **X** |  |

**Key:**

SEQ: SNP allele score in the six cultivars based upon available sequence data

KASP: assay call for the same SNP in the six cultivars

Color codes:

Green: The loci matched between sequence and successful KASP assay

Purple: KASP assay successful but failed to match the sequence

Orange: KASP assay failed in all genotypes.(X: Failed reaction)

No colour: No available sequence data
